# DNA sequence and structural properties as predictors of human and mouse promoters

**DOI:** 10.1016/j.gene.2007.12.011

**Published:** 2008-02-29

**Authors:** Pelin Akan, Panos Deloukas

**Affiliations:** Wellcome Trust Sanger Institute, Hinxton CB10 1SA Cambridge, UK

**Keywords:** TBP, TATA-box binding protein, TSS, transcription start site, CGI, CpG island, WCGI, associated with CpG island, WOCGI, not associated with CpG island, DBTSS, Database of Transcription Start Sites, R, purine, Y, pyrimidine, DPE, downstream promoter element, BRE, TFIIB recognition element, LCG, low CpG count, HCG, high CpG count., Promoter, Human, Mouse, Genome-wide computational analysis, CpG island, TATA-box, DNA bendability, Propeller twist, Nucleosome positioning preference, ATG desert

## Abstract

Promoters play a central role in gene regulation, yet our power to discriminate them from non-promoter sequences in higher eukaryotes is mainly restricted to those associated with CpG islands. Here, we examined *in silico* the promoters of 30,954 human and 18,083 mouse transcripts in the DBTSS database, to assess the impact of particular sequence and structural features (propeller twist, bendability and nucleosome positioning preference) on promoter classification and prediction. Our analysis showed that a stricter-than-traditional definition of CpG islands captures low and high CpG count promoter classes more accurately than the traditional one. We observed that both human and mouse promoter sequences are flexible with the exception of the TATA box and TSS, which are rigid regions irrespective of association with a CpG island. Therefore varying levels of structural flexibility in promoters may affect their accessibility to proteins, and hence their specificity. For all features investigated, averaged values across core promoters discriminated CpG island associated promoters from background, whereas the same did not hold for promoters without a CpG island. However, local changes around − 34 to − 23 (expected position of TATA box) and the TSS were informative in discriminating promoters (both classes) from non-promoter sequences. Additionally, we investigated ATG deserts and observed that they occur in all promoter sets except those with a TATA-box and without a CpG island in human. Interestingly, all mouse promoter sets showed ATG codon depletion irrespective of the presence of a TATA-box, possibly reflecting a weaker contribution to TSS specificity in mouse.

## 1 Introduction

Promoters carry the central regulatory information of genes, therefore their in-depth characterization is vital to understand gene function. At present, there is an immense experimental effort for promoter identification and characterisation using techniques such as gene reporter assays, chromatin immunoprecipitation, oligo-capping, cap analysis of gene expression (CAGE) and 5′ end serial analysis of gene expression (SAGE) (Suzuki et al., 2001; Shiraki et al., 2003; Hashimoto et al., 2004; Kim et al., 2005; Cooper et al., 2006). Such methods generate powerful information towards understanding promoter sequence features as well as their activation mechanisms. One limitation though comes from having only a limited number of tools to mark inactive promoters. *Ab initio* prediction can be an alternative approach to locate promoter regions in a given genome sequence. Several algorithms have been designed to predict promoters and / or transcription start sites (TSSs) (reviewed in Pedersen et al., 1999; Hannenhalli and Levy, 2001; Maston et al., 2006). In a comparative study (Bajic et al., 2004) most prediction programs failed to operate at the genome scale, while predictors such as FirstEF (Davuluri et al., 2001), Eponine (Down and Hubbard, 2002) and CpGproD (Ponger and Mouchiroud, 2002) performed reasonably well for certain promoter sets. Promoters associated with CpG islands (WCGI) were relatively easy to predict; most programs could predict ∼ 80% of those promoters by making one false prediction for every true prediction (Davuluri et al., 2001). No program was able to predict promoters not associated with CpG islands (WOCGI) satisfactorily. For instance, FirstEF (Davuluri et al., 2001) predicted only 5% of such promoters while making 16 false predictions for every true prediction. WCGI promoters are associated with about 60% of human genes and often direct transcription of house-keeping or widely-expressed genes (Antequera, 2003). On the other hand, promoters not associated with CpG islands are generally tissue specific and often associated with a TATA box (Schug et al., 2005; Yamashita et al., 2005). Several studies investigated promoters in terms of base composition analysis, motif finding, comparative genome analysis and tissue-specificity profiles (Babenko et al., 1999; Louie et al., 2003; Aerts et al., 2004; Bajic et al., 2004; Schug et al., 2005). Overall, analysis showed that promoters are GC-rich, enriched with specific transcription binding site motifs depending on their tissue specificity profiles. Specific sequence motifs (TATA box, Initiator, BRE, DPE) within core promoters were found to be present in particular synergetic combinations, suggesting specific modes of transcription initiation depending on the presence and mutual positioning of such elements (Gershenzon and Ioshikhes, 2005). Several additional sequence motifs have also been found in subsets of promoters (FitzGerald et al., 2004; Marino-Ramirez et al., 2004; Xie et al., 2005; Yang et al., 2007).

The limited power of sequence-related information (i.e. base content, transcription factor binding sites, statistical properties of promoter sequences, and comparative genomics approaches) for deriving general signatures for promoters has motivated studies that employ structural properties of DNA in promoter prediction and classification (Pedersen et al., 1998; Gabrielian et al., 1999; Gardiner et al., 2003; Florquin et al., 2005; Kanhere and Bansal, 2005). Indeed, promoter DNA has to possess the required 3D structure to allow DNA-binding events and accommodate alterations in nucleosome positioning in order to allow the start of transcription. TATA boxes and initiator sequences have been found to comprise distinct flexible and rigid sequences compared to other parts of promoters (Pedersen et al., 1998; Babenko et al., 1999; Fukue et al., 2004; Fukue et al., 2005). Studies to measure promoter strength as a function of DNA flexibility using luciferase reporter assays showed that presence of rigid sequences around the expected position of the TATA-box had a positive influence on transcription (Fukue et al., 2004). However, it is important to note that structural properties of DNA are in part a result of the underlying nucleotide content.

Some promoters have been reported to exhibit depletion of ATG triplets around the TSS (Lee et al., 2005). This promoter subclass, called ATG deserts, is typically not associated with a TATA box, independently of the presence or absence of a CpG island (Lee et al., 2005). Lee et al. postulated that such promoters using multiple TSSs would still generate a single peptide starting with a methionine. This mechanism could be valuable especially for TATA-less promoters, which show a diverse distribution of TSS (Suzuki et al., 2001).

In this study, we examined the potential of three DNA structural properties for predicting WCGI and WOCGI promoters, namely propeller twist angle, bendability, and nucleosome positioning preference, and extended previous structural analyses of promoters. We analysed all human and mouse promoters from DBTSS and investigated the details of GC content change and CpG count in WCGI and WOCGI promoters. We also compared different definitions of CpG islands (CGI). Finally, we investigated ATG deserts to estimate their prevalence in the selected promoter sets and their association with presence of the TATA box in human and mouse promoters.

## 2 Materials and methods

### 2.1 Datasets

The datasets of human and mouse promoters in DBTSS (release 5.2.0; (Suzuki, 2001; Yamashita et al., 2006)) were used in this analysis. All 30,964 human promoters in DBTSS were used for analysis, but 940 of 19,023 mouse promoters were not included to the analysis because of repeats and low sequence quality, leaving 18,083 promoters for analysis. We also generated a human and mouse non-promoter sequence set each containing 20,000 random fragments of 1201 bp selected outside exonic regions to avoid the sequence bias of coding DNA.

### 2.2 CpG islands and CpG counts

The program ‘CpG island searcher’ (Takai and Jones, 2002) was used for identification of CGI, which were defined as regions greater than 500 bp in size, with a GC composition ≥ 55%, and an observed/expected CpG ratio of 0.65. The definition used here is more stringent than the original definition (200 bp in size, with a GC composition ≥ 50%, and an observed/expected CpG ratio of 0.60) (Gardiner-Garden and Frommer, 1987) in order to exclude GC-rich Alu-repetitive elements. Of the 30,964 human promoters, 12,229 (40%) were associated with a CGI. Of the 18,083 mouse promoters, 8415 were associated with a CGI. The original definition of CGI classified 17,456 (56%) human transcripts as associated with CGI.

Normalised CpG count was calculated as in (Saxonov et al., 2006), where observed CpG count is divided by expected CpG count calculated as (GC content/2)^2^.

### 2.3 Positional weight matrix

For the analysis, promoter sequences were aligned relative to the TSS. A positional weight matrix was generated by calculating the frequency of each nucleotide for each position in the alignment. The nucleotide frequencies were then normalised by subtracting the expected frequency of each nucleotide at any random site in the human genome. The expected frequencies of nucleotides A, C, G and T at any random site in the human and mouse genome are 29%, 21%, 21% and 29% respectively (Venter et al., 2001; Waterston et al., 2002). Also, uncertainty in the background-subtracted frequencies was calculated assuming that frequencies follow a Poisson distribution.

### 2.4 Structural properties

Bendability is a local feature representing the ability of DNA to bend towards the major groove and was quantified from DNase I digestion studies where every trinucleotide was associated with a score reflecting its bendability towards the major groove (Brukner et al., 1995a). Propeller twist angles are calculated for dinucleotides using measurements from X-ray crystallography of DNA oligomers (el Hassan and Calladine, 1996). Nucleotide position preferences are taken from a study performed on sequences wrapped around nucleosome cores and in circular DNA. Each 32 trinucleotides and their reverse complements are associated with a fractional preference for a position facing out of the histone core (Satchwell et al., 1986). Here, propeller twist, bendability and nucleosome positioning preference scores of all observed di- or trinucleotides in sliding overlapping windows (with one nucleotide step) were calculated and averaged out to calculate the mean score of each di or trinucleotide along promoters and non-promoters as described in Florquin et al. (Florquin et al., 2005). The total mean score of a sequence was calculated by averaging the scores of all di- or trinucleotide windows along the sequence of interest.

### 2.5 Motif finding

We searched all sequences for a degenerate TATA-box motif - HWHWWWWR, excluding HTYTTTWR, CAYTTTWR, MAMAAAAR, and CTYAAAAR (Wobbe and Struhl, 1990). Promoters that contained such a TATA-box motif between − 50 to − 17 bases relative to TSS were considered as containing a TATA-box. Using this definition, there are 4479 (15%) human promoters with a TATA-box, and 85% of those were not associated with a CGI; 21.8% of WOCGI promoters and 5.7% of WCGI promoters contain the TATA-box. In mouse, 2387 (13%) promoters contained a TATA-box and 80% of those were not associated with a CGI.

### 2.6 ATG codon count

As described in Lee et al., every promoter sequence was divided into 100 bp bins and the number of ATG codons was counted; the average count per window is plotted over the sequences (Lee et al., 2005).

## 3 Results

### 3.1 Details of GC content change are different between WCGI and WOCGI promoter sets

**Fig. 1:**
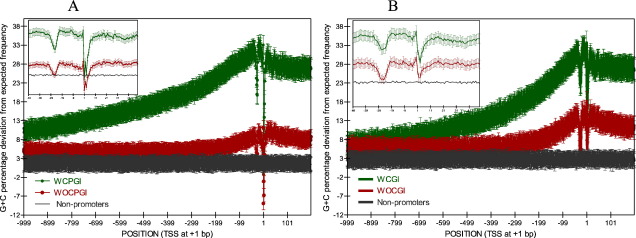
Background subtracted GC content of WCGI, WOCGI and non-promoters in human (A) and mouse (B). The error bars correspond to standard error of the data.

**Fig. 2:**
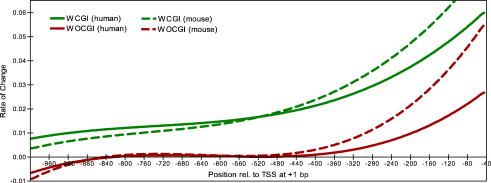
Fourth order polynomial curves were fitted to the background subtracted GC frequency curves of promoter sets (associated or not with CpG islands) between – 1000 to – 40 bp relative to TSS. *R*^2^ values for the fitted curves to promoters associated or not associated with CpG islands are 0.9985 and 0.9668 in humans and 0.9979 and 9844 in mouse respectively.

Promoter sequences are typically GC-rich although the degree of GC-enrichment among promoters is highly variable (Kel-Margoulis et al., 2003; Aerts et al., 2004; Bajic et al., 2004; Saxonov et al., 2006). To study the details of GC content in promoters, we first divided the 30,964 human and 18,083 mouse promoters from DBTSS (5.2.0), corresponding to 14,629 and 13,165 genes respectively, into those associated (WCGI) and those not associated (WOCGI) with a CpG island, using the CGI definition described by Takai and Jones et al., and generated G + C positional weight matrices for each set (Takai and Jones, 2002). WCGI promoters have higher GC content along both the proximal and core region whereas WOCGI promoters show increased GC content only along the extended core region (Fig. 1) in both human and mouse. We also repeated this using the more traditional CGI definition of Gardiner-Garden et al. (Gardiner-Garden and Frommer, 1987), which led to an increased number of WCGI promoters in both organisms (see Section 3.2), but showed essentially the same GC content increase along core WOCGI promoters as when the Takai and Jones definition was used. To understand the details of GC content change along the proximal and core promoter sequences, fourth order polynomial curves were fitted to the GC frequency plots. Differentiation of the fitted curves showed that WOCGI promoters do not show an increase in their GC content up to 400 bp upstream of the TSS while WCGI promoters show a continual increase in their GC content and the rate of change along the sequence increases close to the TSS (Fig. 2). In mouse, GC content showed a higher rate of increase in the proximal promoter region compared to human in both sets of promoters. Particularly WOCGI mouse promoters show a higher rate of increase, as well as higher GC content around the core promoter region (Fig. 1). Recent studies have suggested an accelerated evolution in primate promoter elements possibly due to low effective population sizes which decreases the efficiency of purifying selection, whereas a stronger selective pressure on promoters was observed in the rodent lineage (Keightley et al., 2005; Taylor et al., 2006). With this finding in mind, the observed lower rate of GC content increase in human promoters toward TSS may be an artefact of greater loss of general promoter characteristics (such as increased GC content towards TSS) due to weaker selective constraints in humans compared to mouse. This may have assisted the increased specialisation in human promoters as well as more complex and precise regulatory circuits (Vinogradov and Anatskaya, 2007). Additionally, mouse CGIs tend to be shorter than the corresponding human CGIs but they typically have higher number of CGIs around the TSS, which can also explain the steeper GC gradient in mouse WCGI promoters (Matsuo et al., 1993).

Lastly, we subdivided WCGI and WOCGI promoters with respect to the presence or absence of a degenerate TATA-box motif (Section 2.5), and assessed their GC content. Interestingly, WCGI and WOCGI promoters that do not have a TATA-box still possessed a subtle AT enrichment at the expected position of the TATA box (Fig. S2 A and B). It is important to note that only 0.5% and 2% of WCGI and WOCGI promoters, respectively, contained TATA-box like sequences which are classified as structurally inflexible (Wobbe and Struhl, 1990). The observed subtle AT enrichment may be functional as experimental work has shown that most AT-rich sequences of six base pairs or longer can partially replace TATA-box motif in the proximity of other control elements (Hahn et al., 1989; Singer et al., 1990; Zenzie-Gregory et al., 1993). To further investigate this hypothesis, we examined the AT content of the region around the expected position of a TATA box in both promoter sets (Fig. S3). Less than 8% of the WCGI and less than 1% of the WOCGI promoters did not have any A or T nucleotide in this region (same in human and mouse). Around 15% and 35% of WCGI and WOCGI promoters, respectively, have more than 60% AT nucleotides at the expected position of a TATA-box, while only 5.7% and 21.8% of WPCGI and WOCGI promoters are associated with a TATA-box (Fig. S4). This observation suggests that in addition to a TATA-box motif, the relative enrichment in AT content of the region may play a role in TBP binding in human and mouse promoters.

### 3.2 Stricter definition of CGI captures inherent CpG count patterns more accurately

**Fig. 3:**
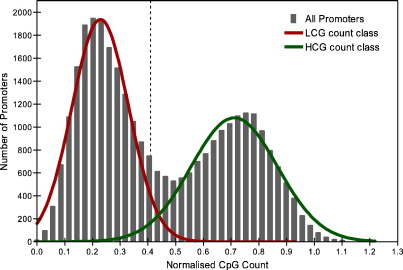
Histogram of normalised CpG count of all promoters and individual Gaussian fits to the overall bimodal distribution (*r*^2^ for bimodal fit is 0.9721). The mean of LCG and HCG count curves are 0.22 ± 0.10 and 0.71 ± 0.16 respectively. The point for which an equal fraction of promoters are misidentified for both distributions (0.41) is taken as the threshold to separate promoters into LCG and HCG count, and the relative distribution of the promoters in LCG and HCG count classes are 55% and 45% respectively.

**Fig. 4:**
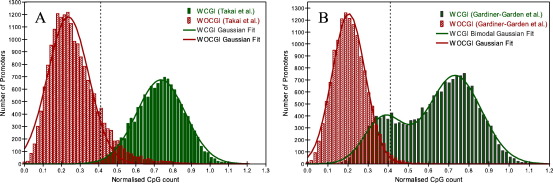
(A) Normalised CpG count histograms of WCGI and WOCGI promoters classified using the definition from Takai et al (Takai and Jones, 2002). Distribution of WCGI and WOCGI promoters are approximated by two Gaussian curves with means of 0.24 ± 0.11 and 0.74 ± 0.13. (B) Normalised CpG count histograms of WCGI and WOCGI promoters classified using the CGI definition from Gardiner-Garden et al (Gardiner-Garden and Frommer, 1987). Distribution of WOCGI promoters is approximated by a Gaussian curve with mean of 0.20 ± 0.08, whereas distribution of WCGI promoters showed a bimodal distribution with two Gaussian curves with means of 0.38 ± 0.10 and 0.73 ± 0.14. Dotted lines correspond to the threshold (0.41) to separate promoters with low and high CpG count.

Saxonov et al. employed CpG counts to classify human promoters into sets with distinct CpG count distributions, one with low and one with high mean (Saxonov et al., 2006). We applied the same classification scheme to our dataset and found a bimodal distribution of normalised CpG count (Fig. 3), approximated by two Gaussian curves with means of 0.22 ± 0.10 and 0.71 ± 0.16. The point at which an equal fraction of promoters are misidentified for both distributions (0.41) was taken as the threshold to separate promoters into low and high CpG (LCG and HCG count), giving 55% and 45% of promoters in LCG and HCG class respectively. The normalized CpG count of WCGI and WOCGI promoters was also calculated and the two sets roughly correspond to the LCG (with a mean of 0.73 ± 0.13) and HCG (with a mean of 0.27 ± 0.13) count classes respectively (Fig. 4A). Based on the CGI classification of Gardiner-Garden et al. (Gardiner-Garden and Frommer, 1987), we found that the mean normalized CpG count of WCGI and WOCGI promoters is 0.63 ± 0.19 and 0.21 ± 0.08 respectively. Interestingly, WCGI promoters showed a bimodal CpG count distribution (with means of 0.38 ± 0.10 and 0.73 ± 0.14) with a subset of them falling into the LCG count class (Fig. 4B). The fact that the promoter sets generated by the stricter CGI definition overlap more accurately with the inherent CpG count classes of the complete promoter set suggest that the stricter definition might be a more biologically reliable classification scheme.

Although the means of the low and high CpG count classes agree between our study and Saxanov et al., the relative number of promoters within each set differs. Dissimilarities between the studies include the use of 1000 bp upstream and 200 bp downstream of the TSS (this study) versus 1500 bp upstream and 1500 bp downstream by Saxonov et al. We calculated normalised CpG count for the same window, but this did not change the relative distribution of promoters. The other significant difference between the two studies is the datasets used. We included experimentally verified promoter sequences obtained from 166 cDNA libraries and 1,548,357 confirmed full-length cDNAs. Saxanov et al. used promoter regions of validated transcript annotations in human genome build 16 (July 2003) (Pruitt et al., 2007). It is therefore possible that our dataset contains a much higher number of alternative and tissue-specific promoters as a result of the high number of cDNA libraries used. Typically, such promoters are not associated with CpG islands and expected to belong to the LCG count class.

### 3.3 DNA structural properties are more common in GC-rich promoters

Promoter sequences are docking regions for assembly of the transcription initiation complex and therefore expected to possess distinct structural abilities to allow functional protein binding events and nucleosome positioning. Hence it is necessary to investigate promoter sequences in higher dimensions than the sequence alone, which accounts only for the adjacency of bases. Here, we examined three structural properties of promoter sequences: bendability, propeller twist and nucleosome positioning, and assessed their power to distinguish promoters from non-promoter sequences.

#### 3.3.1 Propeller twist

**Fig. 5:**
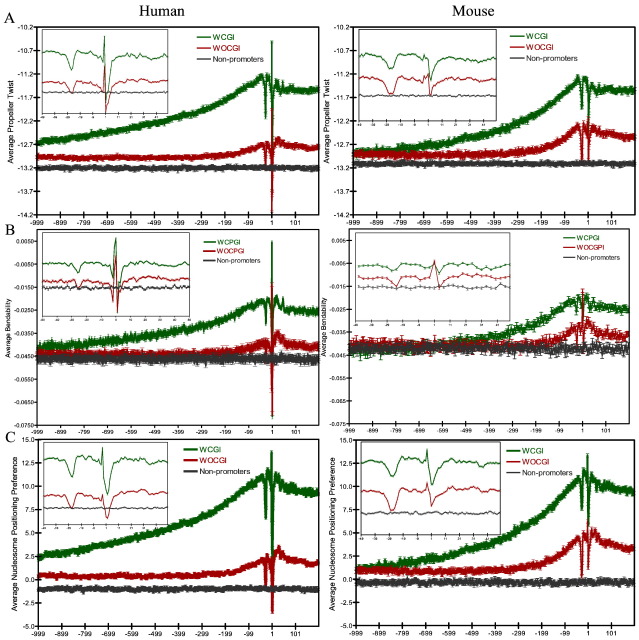
Sequence and structural profiles of WCGI, WOCGI and non-promoters in human (left) and mouse (right). (A) Average propeller twist of each dinucleotide window (B) average bendability of each trinucleotide window (C) Average nucleosome positioning preference of each trinucleotide window along all sequences. Inset figures shows profiles between 50 bp upstream and downstream of TSS.

The bases of a DNA base pair are rarely co-planar, but rather twisted with respect to each other. The dihedral angle between the planes of base pairs is called propeller twist and it is a measure of helix rigidity since it has been shown to be inversely related to the rigidity of the DNA helix in crystals (el Hassan and Calladine, 1996). The propeller twist enhances the stacking of the bases on one strand and therefore increases the stability of the helix meaning that regions with high propeller twist angles (large negative) would correspond to rigid areas, whereas flexible regions would have low propeller twist angles. To compare rigidity profiles, we calculated propeller twist angles along WCGI and WOCGI promoters and non-promoter sequences in both organisms. The propeller twist angles used here were obtained from X-ray crystallography data (el Hassan and Calladine, 1996) for all possible dimers and according to which the most flexible dimer is GG/CC (− 8.11) and the most rigid dimer is AA/TT (− 18.11). In both organisms, both promoter sets showed increased flexibility towards the TSS, but were considerably rigid at the expected positions of the TATA-box and TSS (Fig. 5A). Promoters not containing a consensus TATA-box also exhibited some rigidity at the − 34 to − 23 region (Fig. S5) plus an increase in propeller twist angles downstream of the TSS. WCGI promoters were more flexible than WOCGI promoters. The profiles of propeller twist angle in human and mouse differed at the TSS, with human TSSs seemingly accommodating a more rigid structure compared to mouse. To investigate this difference, we calculated dimer frequencies at the TSS in WCGI and WOCGI promoters and divided each figure to the dimer frequency in non-promoter sequences (Fig. S1). Rigid dimers were overrepresented in human TSSs but not in mouse.

**Fig. 6:**
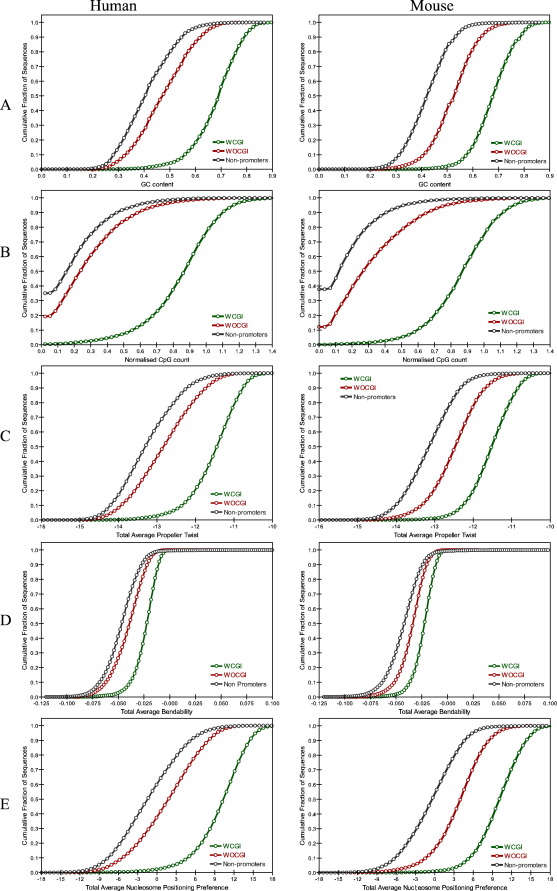
Cumulative histograms of total (A) GC content (B) normalised CpG count (C) propeller twist (D) bendability and (E) nucleosome positioning preference within 100 bp upstream and 50 bp downstream of TSS of promoter sequences and 150 bp non-promoter sequences.

Next, we calculated the average propeller twist of the 100 bp upstream and 50 bp downstream regions of TSSs and generated cumulative histograms to investigate differences between the sets (Fig. 6C). Human and mouse core promoters associated with a CGI exhibited more flexible structures with less negative average propeller twist angle values compared to background. However, the mean propeller twist angle along WOCGI core promoters was rather similar (but less in mouse) to non-promoters. WCGI promoters have a distinct propeller twist angle compared to background sequences. Therefore mean propeller twist angle may not have enough discriminatory power for recognizing WOCGI promoters over background but local variation at the expected position of a TATA box and TSS might mark core promoter sequences irrespective of their association with a CGI.

#### 3.3.2 Bendability

DNA bending induced by protein binding events plays a critical role in control of gene expression by a promoter. The extent of bending depends not only on the protein, but also on the structural flexibility of DNA — which is intrinsic to the DNA molecule. Proteins generally induce bends towards the major groove (with positive roll angles) irrespective of their binding site on the DNA (Brukner et al., 1995b). DNase I enzyme activity strongly depends on the inherent bendability of its cognate site, which makes it an ideal tool for investigating intrinsic bending abilities of DNA (Travers, 1993). Brukner et al. examined DNase I activity in relation to its binding site base content and derived parameters for 32 trinucleotides corresponding to their tendency to bend towards the major groove (Fig. S8) (Brukner et al., 1995a). We employed these parameters to locate bendable regions towards the major groove along promoters as well as non-promoters. WCGI promoters showed higher bendabilities (more flexible) compared to WOCGI promoters (Fig. 5B). Both promoter sets have lower bendability towards the major groove at the expected position of a TATA-box, which is not expected since TATA-box binding protein (TBP) induces a bend towards the major groove (Lilley, 1995). On the other hand, the comparison of X-ray crystallography data from DNA-protein structures with trinucleotide bendability parameters reveals a disagreement only in case of TBP binding (Brukner et al., 1995a) most likely due to an unusual conformation of the TBP-DNA complex compared with other protein-DNA structures. Therefore, the bendability profile at the expected position of TATA-box may not be very informative to describe *in vivo* DNA conformation requirements for TBP binding. Note that 21.8% of WOCGI promoters contain a degenerate TATA-box motif in humans, but only 5.7% of WCGI promoters have it. However, the rigid profile at the expected position of a TATA-box is present irrespective of the presence of the motif (Fig. S6).

Interestingly, TSS also showed a rigid profile in both WOCGI and WCGI promoters with decreased ability to bend towards the major groove. Detailed conformation of DNA at the TSS within the transcription initiation complex is not clear in human and mouse. However, structure resolution of initiator binding protein 39 kDa (IBP39) in a primitive eukaryote, *Trichomonas vaginalis*, has revealed insights into DNA conformations at TSS (Schumacher et al., 2003). DNA bound to IBP39 was smoothly bent by 20°, with no major distortions and its major groove was widened by 3° compared to B-DNA. This finding is in agreement with our observations since a conformation with less bending ability towards the major groove will be more suitable for major groove widening.

WOCGI promoters showed similar bendability profile as that of non-promoter sequences up to circa 100 bp upstream of the TSS. In order to determine whether the total average bendability scores differ significantly between promoter classes and non-promoter sequences within the core promoter region, the relative cumulative histograms of average total bendability were generated for 100 bp downstream and 50 bp upstream of the TSS (Fig. 6D). WCGI promoters have an overall high average total bendability whereas those of WOCGI and non-promoters are similar. These findings suggest that the average total bendability values could be used to discriminate WCGI promoters, but not WOCGI promoters, from the background.

#### 3.3.3 Nucleosome positioning

Transcriptional activity and chromatin structure are tightly coupled. Experimental studies have shown that nucleosomes are not randomly positioned but rather assembled preferentially at sites that favour DNA bending, which are called nucleosome positioning sequences. Moreover, there is a statistical preference for rotational positioning of DNA around the histone octamer such that AT-rich segments have the DNA minor groove facing the octamer, while the minor groove of GC-rich regions faces away from the protein (Travers, 1993). Here, we employed a trinucleotide model which gives the fractional preference of all trinucleotides for a position where its minor groove is facing away from the nucleosome, to investigate intrinsic nucleosome positioning preference along promoter sequences (Fig. S9) (Satchwell et al., 1986). In this model, positive values denote regions where the minor groove faces away from the histones and negative values denotes regions where the minor groove faces towards the histone core. The nucleosome positioning profiles of human and mouse promoters show an elevated number of sequences especially in WCGI promoters that prefer to face away from the nucleosome (exposing their minor groove) (Fig. 5C). Surprisingly, the expected position of TATA-box seems to prefer to be facing towards the histone core, which is not expected since TBP binds to the minor groove. However, detailed structural analysis on TBP-TATA box binding reveals that TBP binds to its cognate site when its minor groove is facing towards the histone core (Imbalzano et al., 1994). A model of TBP binding to a TATA box motif whose minor groove is facing out shows that the TBP-induced bend in DNA is much higher than the actual bend obtained from X-ray crystallography data (Imbalzano et al., 1994). Therefore, although the nucleosome positioning profile at this position does not conform to expectations, it appears to provide relevant conformation for TBP binding (Imbalzano et al., 1994). Additionally, this particular nucleosome positioning at the expected position of TATA-box is also observed in promoters without a consensus TATA-box (Fig. S7).

Both human and mouse TSSs preferred sequences that are facing towards the histone, hiding their minor groove, hence exposing their major groove. The initiation motif found at the TSS is another point of contact with the transcription initiation complex, specifically with TBP-associated factor 1 (TAF1) and TBP-associated factor 2 (TAF2) (Chalkley and Verrijzer, 1999). Although the structure of TAF2 is not yet known, resolved structure of TAF1 at 2.1 Å shows that it is an all-alpha protein with multiple domains (Jacobson et al., 2000). Many transcription factors use their alpha helices to bind DNA by positioning them into the major groove of DNA (Draper, 1995). Therefore, nucleosome positioned exposing the major groove might assist TAF1 binding using its alpha helices at TSS. Additionally, the helix-turn-helix DNA binding motif of IBP39 shows homology with TAF2 and its binding causes a major groove widening at TSS (Schumacher et al., 2003). This also supports our observations since such nucleosome positioning preference at TSS allows an accessible major groove, more available to protein-induced deformations.

We also generated average total nucleosome positioning preferences for core promoters (100 bp upstream and 50 bp downstream of TSS) and their cumulative histograms for WCGI and WOCGI promoters and non-promoters in both organisms (Fig. 6E). As for bendability, average total nucleosome positioning values did not differ significantly between WOCGI promoters and non-promoter sequences but did for the WCGI set.

### 3.4 ATG codon depletion appears to be more prominent in CpG-island associated promoters

**Fig. 7:**
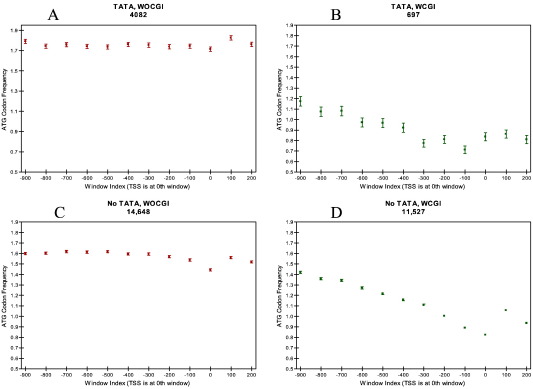
The ATG codon frequency per 100 bp windows in humans on (A) WOCGI promoters with TATA-box, (B) WCGI promoters with TATA-box, (C) WOCGI promoters with no TATA-box and (D) WCGI promoters with no TATA-box.

**Fig. 8:**
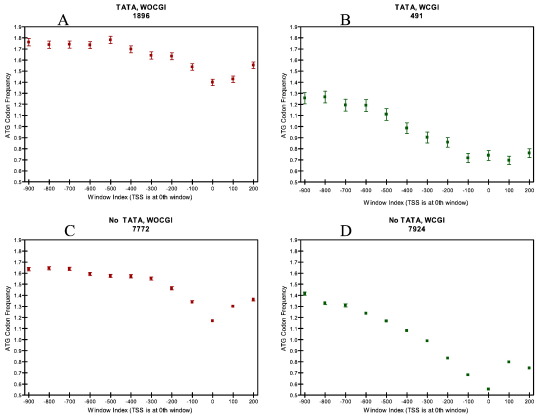
The ATG codon frequency per 100 bp windows in mouse on (A) WOCGI promoters with TATA-box, (B) WCGI promoters with TATA-box, (C) WOCGI promoters with no TATA-box and (D) WCGI promoters with no TATA-box.

It has been reported that ATG trinucleotides are underrepresented ∼ 1 kb upstream and downstream of the TSS especially in promoters lacking a consensus TATA-box (Lee et al., 2005). This finding correlates well with the finding that TATA-less promoters have multiple start sites compared to promoters containing a TATA-box motif but still give rise to a single protein (Suzuki et al., 2001). We searched the DBTSS human and mouse promoter datasets for ATG deserts. Human promoters not carrying a TATA box showed ATG codon depletion around the TSS irrespective of their association with a CGI (Fig. 7 C and D). Among the human promoters carrying a TATA-box, WOCGI promoters did not show any ATG depletion although WCGI promoters showed a slight depletion (Fig. 7 A and B). Moreover, mouse promoters showed ATG codon depletion irrespective of the promoter's association with a CGI or TATA-box, which might suggest a relatively weaker contribution of TATA boxes on TSS specificity compared to humans (Fig. 8). ATG codon depletion appears to be a mechanism for the prevention of producing multiple proteins from loci with multiple TSSs. It might be that the positive correlation between the presence of a TATA-box and the presence of a specific TSS is not as strong in mouse promoters, although one cannot exclude the existence of other sequence motifs effecting TSS specificity.

## 4 Discussion

We analysed the promoter sequences of 30,964 human transcripts (14,629 genes) and 18,083 mouse transcripts (13,165 genes) in DBTSS (5.2.0). As expected, GC content is significantly higher in promoter than non-promoter sequences (intergenic DNA and introns) and, more importantly, the change in GC content along the promoter is not uniform with its core segment showing the highest increase. On the other hand, within these segments of high GC content there are AT enriched regions such as the expected positions of the TATA box and the TSS, and these regions presented distinct structural signatures. Consistent with previous findings, we observed the pyrimidine-purine (YR) step to be enriched in human TSSs (Fig. S1). DNA structural studies have shown the YR dimer to be the most flexible step (Olson et al., 1998), and sharp bends in protein — DNA interactions are mostly accommodated by this step (Suzuki and Yagi, 1995; Werner et al., 1996; Dickerson, 1998) which also facilitates DNA loop formation (Barber and Zhurkin, 1990). However, the fact that there is less YR enrichment in mouse TSSs might mean that the initiation assembly in this species may require different DNA structural conformations. Alternatively, the YR dimer may not be required for a particular structure in the transcription assembly but operate on a sequence-specific level which is not conserved in mouse.

We found that promoter sequences are flexible and that WCGI promoters displayed a higher flexibility along their extended and core sections whereas WOCGI promoters were flexible (above background levels) only around the core region. The GC gradient along core promoters was a property even of WOCGI promoters that belong to the LCG count class, which shows that high GC content is not a result of the CGI definition used to classify WOCGI and WCGI promoters. House-keeping or widely-expressed genes are mostly genes with CGI (Larsen et al., 1992; Ponger et al., 2001) and are therefore expected to be active most of the time and accessible to proteins in many tissues. Promoter sequences are flexible assisting accessibility to proteins and hence increasing the likelihood of transcription. However, WOCGI promoters may prefer sequences with less intrinsic flexibility to achieve restricted activity that is they would be exposed to a smaller number of factors (transcription factors, chromatin structure modifiers). The nucleosome locations relative to the DNA sequence is also important; that is to say nucleosome occupation and its positioning preference (translational or rotational) will have a direct effect on DNA accessibility (Sewack and Hansen, 1997; Chen and Yang, 2001). Therefore, it is reasonable to state that the flexible and accessible structure of promoters will facilitate binding events to varying degrees but the chromatin environment is an additional determinant governing actual binding events.

We also observed a strong correlation between GC content and structural features (Figs. 1 and 5) which might be the result of either the dominant effect of base content on structural parameters, or global DNA structures partially dictating the overall GC content. However, the fact that the observed structural features of promoters conform to expectations supports the latter option.

When looking at structural features on a genomic scale, and averaging along long stretches of DNA, one may not be able to distinguish between promoter and non-promoter sequences, especially for the WOCGI promoter class (Fig. 6). However, since local sequence and structural changes at the TSS and − 34 to − 23 region are conserved in promoters irrespective of their GC content, they might be used to mark WOCGI promoters as well.

Lastly, we find that the ATG triplet is depleted in nearly all human promoter sets with the exception of WOCGI promoters containing a TATA box, a subclass comprising 13.2% of human promoters. Interestingly, the depletion pattern between WCGI promoters with and without a TATA box differs slightly. WCGI promoters without a TATA box showed the highest level of depletion 100 bp upstream of the TSS, whereas WCGI promoters with TATA box showed the highest level of depletion 200 bp upstream of the region. In this case, it might be interesting to investigate the coding potential of first exons in each set and whether a slight increase in ATG count in WCGI promoters with TATA box is related to a coding first exon.

## Appendix A Supplementary data

Fig. S1 Overrepresented dimers at TSS in human and mouse promoters, calculated by normalising the frequency of a dimer by dividing it to its frequency in nonpromoter sequences. Values below each dimer corresponds to their propeller twist angles.

Fig. S2 Human promoters associated or not associated with CpG islands are divided into two groups based on the presence or absence of a degenerate TATA box. (A) Percentage deviation in G + C content from its expected frequency of CpG island associated promoters that contain or do not contain a degenerate TATA-box (dTATA). (B) Percentage deviation in G + C content from its expected frequency of promoters not associated with CpG islands that contain or do not contain a degenerate TATA-box.

Fig. S3 AT content of promoters associated or not associated with CpG islands and non-promoter sequences between 79 bp upstream and 19 bp downstream of the TSS were analysed. The region of interest in divided into 14 bins and each bin size is 7 bp. Vertical axis corresponds to the percentage of promoters with no A or T nucleotides within the windows of seven bp between 79 bp upstream and 19 bp downstream of TSS.

Fig. S4 Vertical axis corresponds to the percentage of the promoters that have an AT% equal or higher than 60% in a bin in WCGI and WOCGI human promoter sets.

Fig. S5 Average propeller twist angles of human promoters containing TATA-box (4479 promoters) and promoters without a TATA-box at the expected position of TATA-box.

Fig. S6 Average bendability profiles of human promoters containing TATA-box and promoters without a TATA-box (at the expected position of TATA-box).

Fig. S7 Average nucleosome positioning preferences of human promoters containing a TATA-box and promoters without a TATA-box at the expected position of TATA-box.

Fig. S8 Minor and major grooves are shown in a simplistic DNA sketch. Bendability values attained to each triplet by Brukner et al. represents DNA intrinsic ability to bend towards major groove (shown by red arrows).

Fig. S9 A rough schematic explanation of nucleosome positioning preference feature. Sequences with high nucleosome positioning preference tends to have their minor groove facing the histone octamer, whereas sequences with low nucleosome positioning preference tends to have their minor groove away from the nucleosome.
