# Supplementary material for: DNA sequence and structural properties as predictors of human and mouse promoters
Source: Gene. 2008 Feb 29;410(1-2):165–76. doi: 10.1016/j.gene.2007.12.011 (PMC2672154; doi:10.1016/j.gene.2007.12.011)
Supplement: Fig. S1 — Overrepresented dimers at TSS in human and mouse promoters, calculated by normalising the frequency of a dimer by dividing it to its frequency in nonpromoter sequences. Values below each dimer corresponds to their propeller twist angles. [file mmc1.pdf]

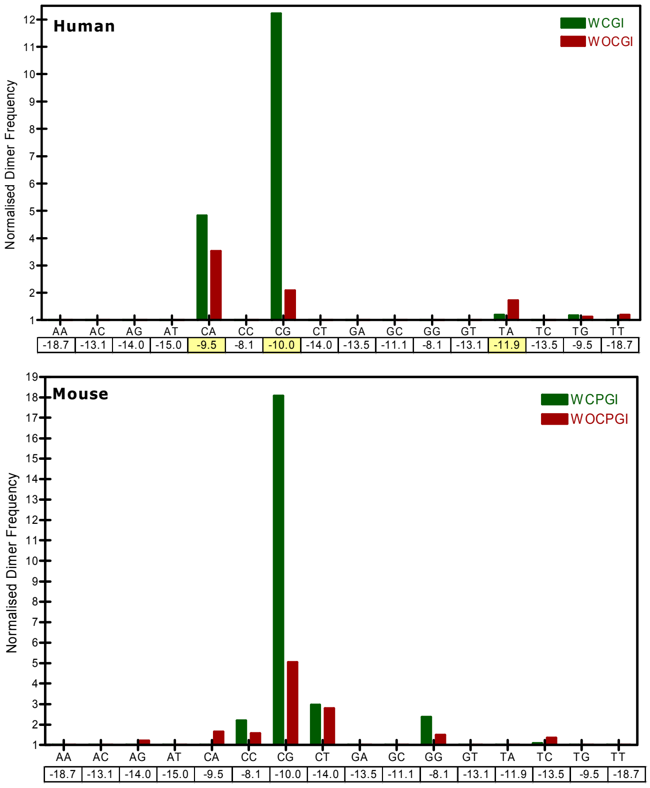

Figure S1 Overrepresented dimers at TSS in human and mouse promoters, calculated by normalising the frequency of a dimer by dividing it to its frequency in non-promoter sequences. Values below each dimer corresponds to their propeller twist angles.

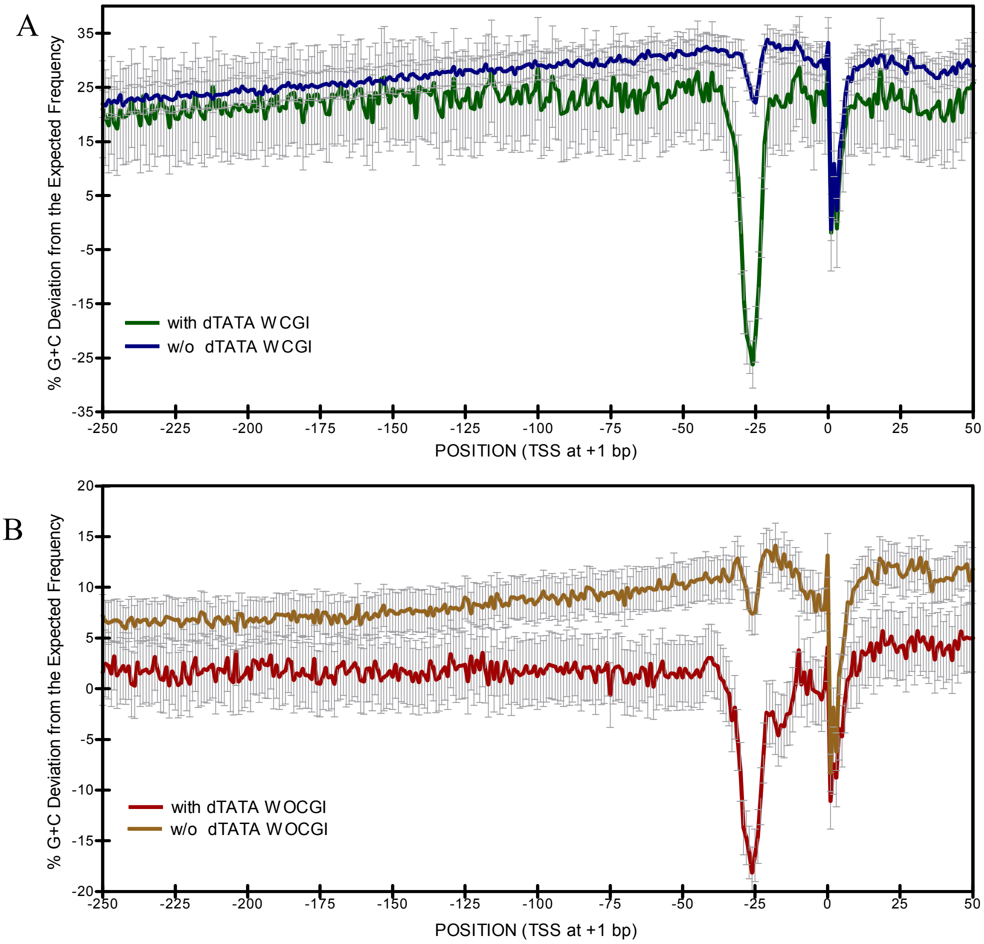

Figure S2 Human promoters associated or not associated with CpG islands are divided into two groups based on the presence or absence of a degenerate TATA box. (A) Percentage deviation in G+C content from its expected frequency of CpG island associated promoters that contain or do not contain a degenerate TATA-box (dTATA). (B) Percentage deviation in G+C content from its expected frequency of promoters not associated with CpG islands that contain or do not contain a degenerate TATA-box.

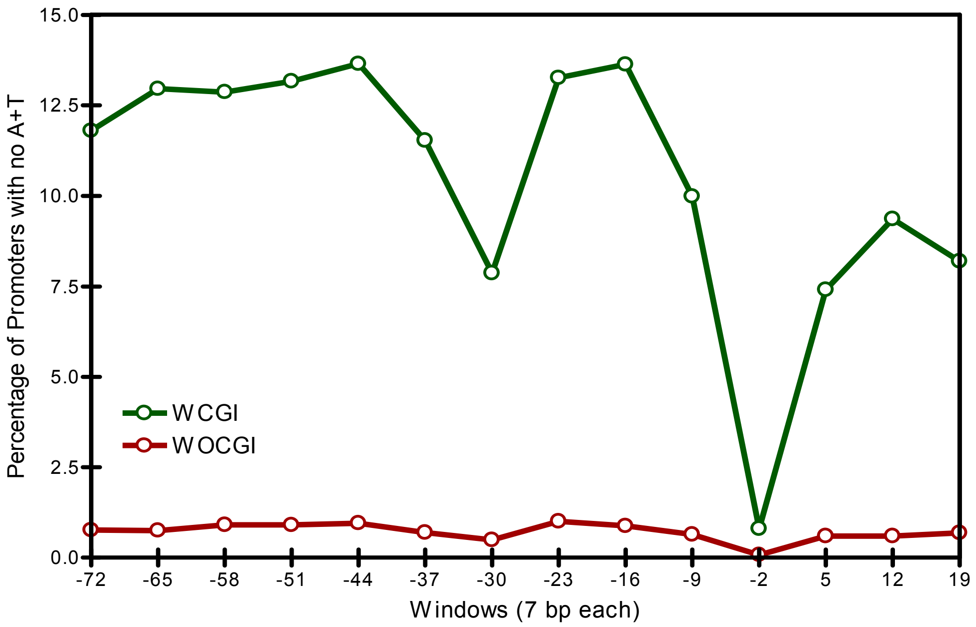

Figure S3 AT content of promoters associated or not associated with CpG islands and non-promoter sequences between 79 bp upstream and 19 bp downstream of the TSS were analysed. The region of interest in divided into 14 bins and each bin size is 7 bp. Vertical axis corresponds to the percentage of promoters with no A or T nucleotides within the windows of seven bp between 79 bp upstream and 19 bp downstream of TSS.

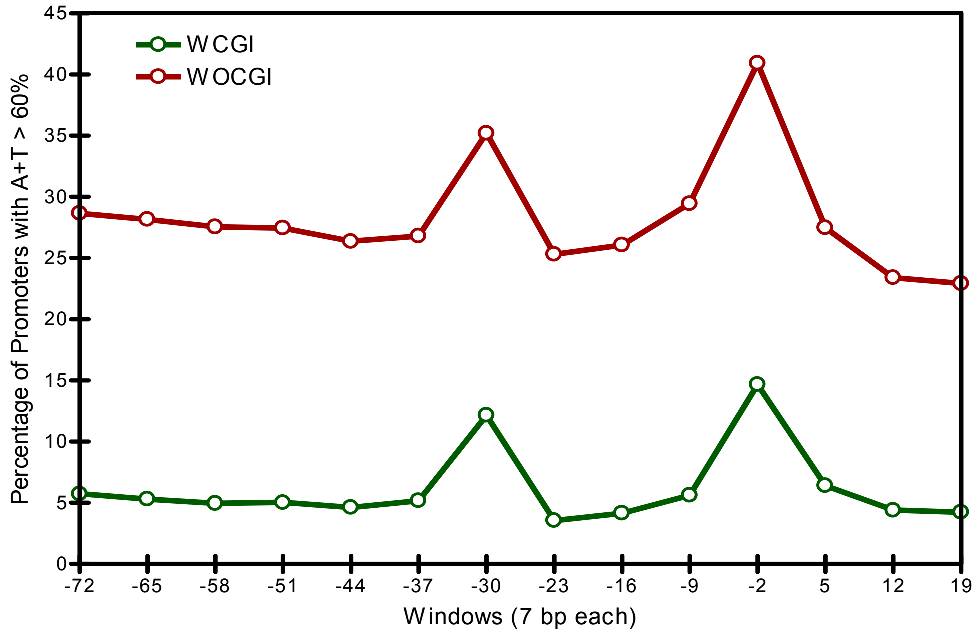

Figure S4 Vertical axis corresponds to the percentage of the promoters that have an AT% equal or higher than 60% in a bin in WCGI and WOCGI human promoter sets.

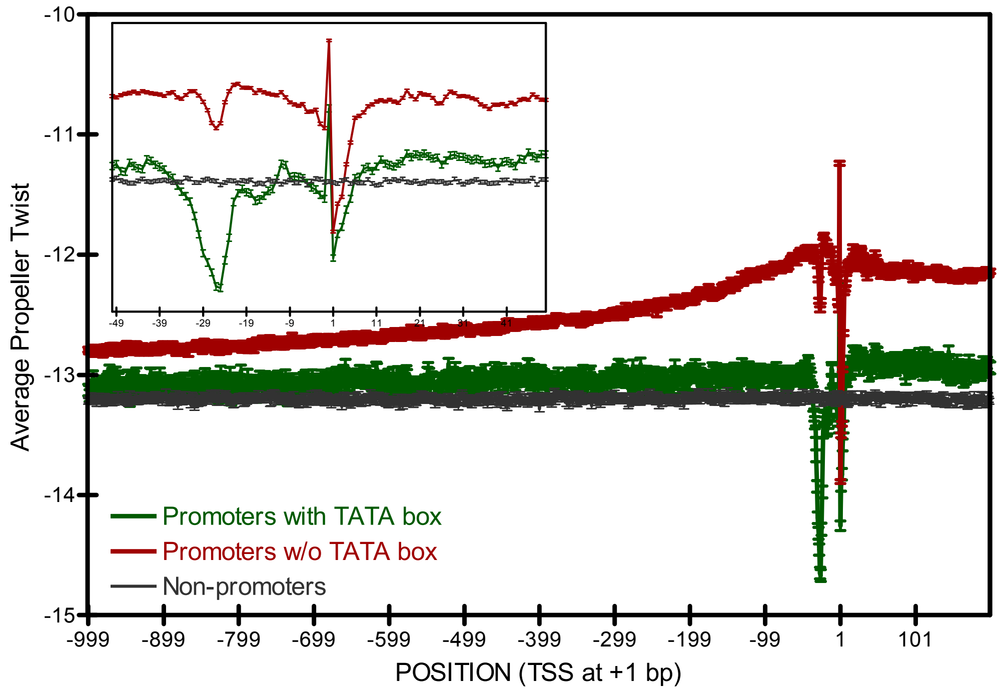

Figure S5 Average propeller twist angles of human promoters containing TATA-box (4479 promoters) and promoters without a TATA-box at the expected position of TATA-box.

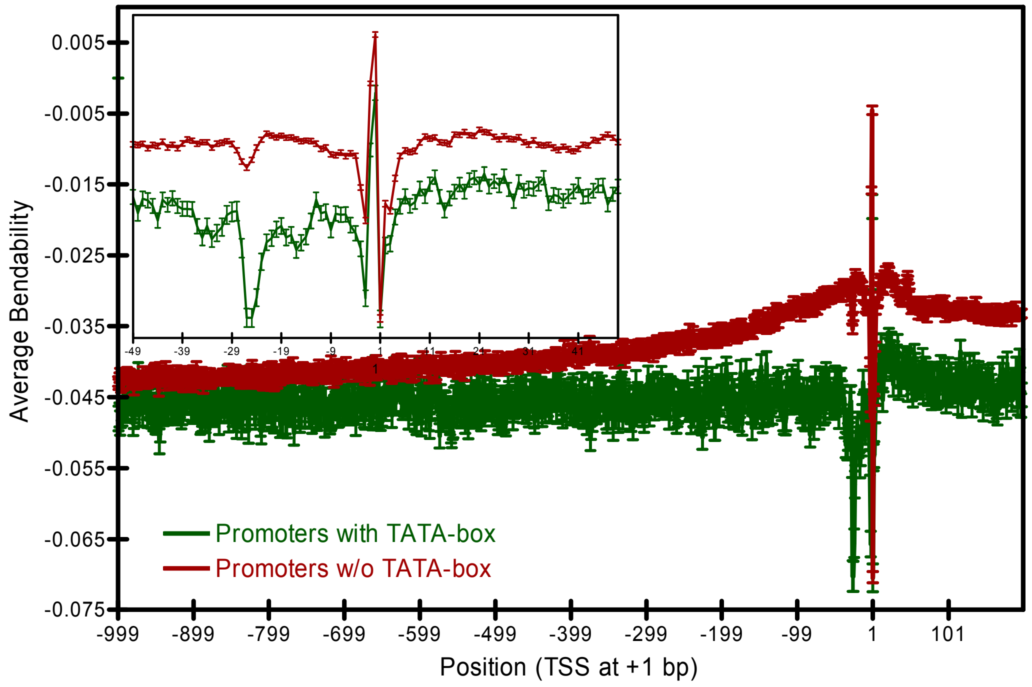

Figure S6 Average bendability profiles of human promoters containing TATA-box and promoters without a TATA-box (at the expected position of TATA-box.

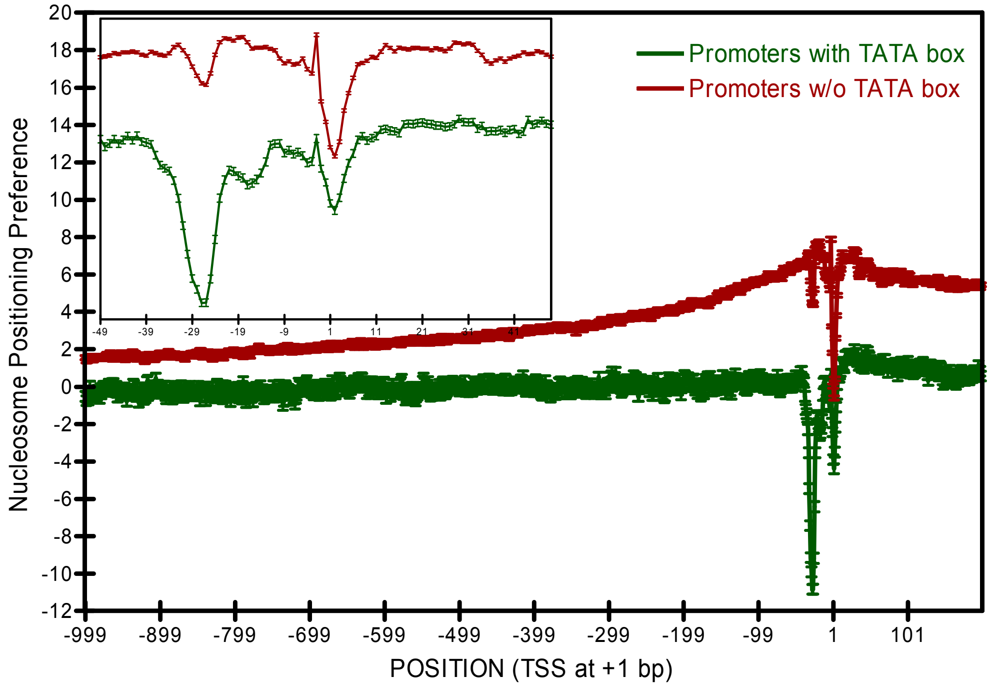

Figure S7 Average nucleosome positioning preferences of human promoters containing a TATA-box and promoters without a TATA-box at the expected position of TATA-box.

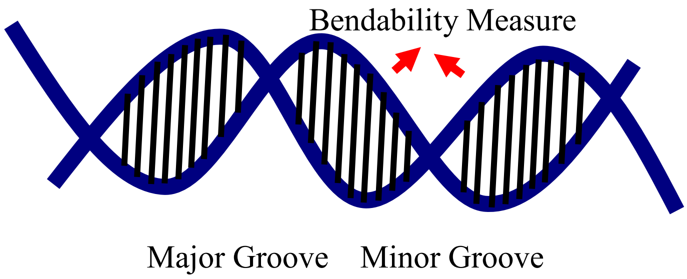

Figure S8 Minor and major grooves are shown in a simplistic DNA sketch. Bendability values attained to each triplet by Brukner et al. represents DNA intrinsic ability to bend towards major groove (shown by red arrows).

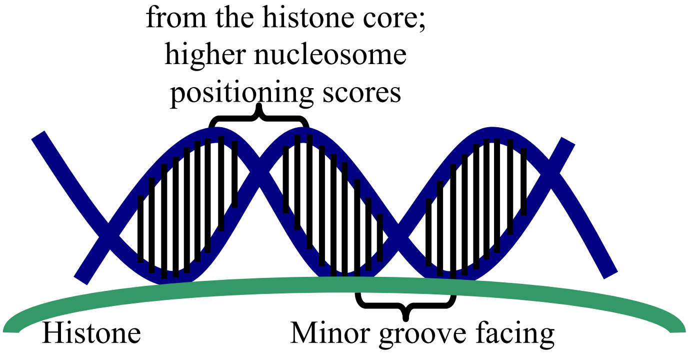

Figure S9 A rough schematic explanation of nucleosome positioning preference feature. Sequences with high nucleosome positioning preference tends to have their minor groove facing the histone octamer, whereas sequences with low nucleosome positioning preference tends to have their minor groove away from the nucleosome.
